# Non-Monotonic Survival of *Staphylococcus aureus* with Respect to Ciprofloxacin Concentration Arises from Prophage-Dependent Killing of Persisters

**DOI:** 10.3390/ph8040778

**Published:** 2015-11-17

**Authors:** Elizabeth L. Sandvik, Christopher H. Fazen, Theresa C. Henry, Wendy W.K. Mok, Mark P. Brynildsen

**Affiliations:** 1 Department of Chemical and Biological Engineering, Princeton University, Princeton, NJ 08544, USA; E-Mails: sandvikliz@gmail.com (E.L.S.); chrisfazen@gmail.com (C.H.F.); wmok@princeton.edu (W.W.K.M.); 2 Department of Molecular Biology, Princeton University, Princeton, NJ 08544, USA; E-Mail: th5@princeton.edu; 3 Rutgers Robert Wood Johnson Medical School, Piscataway, NJ 08854, USA

**Keywords:** fluoroquinolone, persistence, prophage induction, *S. aureus*

## Abstract

*Staphylococcus aureus* is a notorious pathogen with a propensity to cause chronic, non-healing wounds. Bacterial persisters have been implicated in the recalcitrance of *S. aureus* infections, and this motivated us to examine the persistence of *S. aureus* to ciprofloxacin, a quinolone antibiotic. Upon treatment of exponential phase *S. aureus* with ciprofloxacin, we observed that survival was a non-monotonic function of ciprofloxacin concentration. Maximal killing occurred at 1 µg/mL ciprofloxacin, which corresponded to survival that was up to ~40-fold lower than that obtained with concentrations ≥ 5 µg/mL. Investigation of this phenomenon revealed that the non-monotonic response was associated with prophage induction, which facilitated killing of *S. aureus* persisters. Elimination of prophage induction with tetracycline was found to prevent cell lysis and persister killing. We anticipate that these findings may be useful for the design of quinolone treatments.

## 1. Introduction

*Staphylococcus aureus* is a dangerous, Gram-positive pathogen that is frequently associated with nosocomial infections [1,2,3]. *S. aureus* infections can be difficult to cure because many subspecies are resistant to one or more antibiotics (e.g., methicillin-resistant *S. aureus* (MRSA), multidrug-resistant (MDR) *S. aureus*) [3,4] and both planktonic and biofilm populations of *S. aureus* contain high levels of persisters [5,6,7,8,9]. Persisters are phenotypic variants within bacterial populations that have temporary abilities to tolerate high antibiotic concentrations, and their capacity to resume growth following the conclusion of antibiotic therapy has led to the hypothesis that they are important contributors to chronic infections [10,11,12,13]. Persisters are distinct from antibiotic-resistant mutants because they are not endowed with heritable, genetic traits that enable them to grow in antibiotic concentrations that exceed the minimum inhibitory concentration (MIC) of antibiotic-sensitive strains. Rather, they are genetically identical to their kin that die from treatment, and their tolerances have largely been attributed to temporary inhibition of essential cell processes, which reduces corruption of antibiotic primary targets [13,14]. Such dormant states can be achieved through accumulation of toxins from toxin-antitoxin (TA) modules, of which *S. aureus* has several [15,16,17,18,19]. Dormant cells exhibit reduced rates of cell death in the presence of antibiotics, which manifests as biphasic killing kinetics when survival is plotted on a log-scale as a function of time [20,21,22].

Here, we investigated the persistence of *S. aureus* NCTC 8325 to ciprofloxacin. Interestingly, we observed that survival of *S. aureus* was at a minimum at 1 µg/mL ciprofloxacin and increasing the concentration further led to increased survival. At ≥ 5 µg/mL ciprofloxacin, biphasic killing kinetics were observed, and the abundance of survivors reached a plateau with regard to ciprofloxacin concentration, confirming that those cells were persisters. On the basis of previous studies that investigated the impact of quinolones on transcription, translation, and prophage induction [23,24,25,26,27], we hypothesized that the vast majority of persisters were killed at 1 µg/mL ciprofloxacin due to prophage induction. To test this hypothesis, we measured cell lysis, phage titers, bacterial survival, and ciprofloxacin-dependent induction of protein production, and further, we blocked prophage induction with tetracycline. We discovered that persister killing was dependent on prophage induction and anticipate that this knowledge will prove useful for the design of quinolone treatments for *S. aureus* infections.

## 2. Results and Discussion

### 2.1. Survival of S. aureus is Non-monotonic with Respect to Ciprofloxacin Concentration

**Figure 1:**
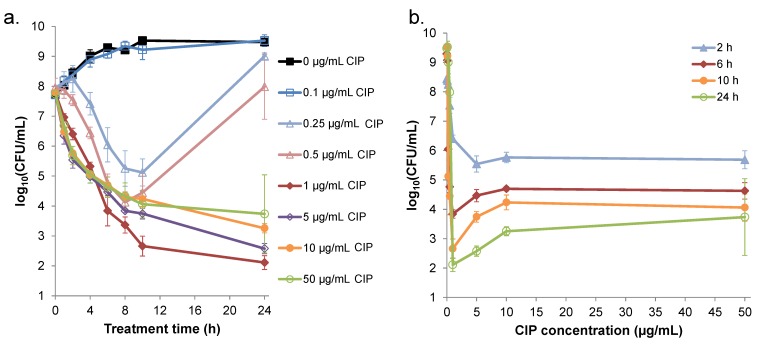
Ciprofloxacin (CIP) treatment of *S. aureus* cultures. *S. aureus* NCTC 8325 was grown in LB for 4 h prior to treatment with 0–50 µg/mL ciprofloxacin. At designated time points, samples were washed to remove ciprofloxacin, serially diluted, plated on LB agar, and incubated overnight to enumerate colony-forming units (CFUs). Survival data are plotted *versus* (**a**) treatment time and (**b**) CIP concentration. Data points are the mean of the log_10_(CFU/mL) across three to four replicate experiments and error bars indicate standard error of the mean (SEM), except for the 24 h time point of 50 µg/mL CIP, which was only replicated twice.

Persisters are enumerated from antibiotic-treated cultures that display biphasic killing kinetics, where the second regime, which exhibits a reduced rate of cell death, comprises persisters [12,13,28,29]. To perform these experiments, an antibiotic concentration high enough to kill any spontaneously-generated mutants must be used, and the duration of treatment to reach the second regime of killing must be identified. Therefore, to identify treatment conditions to quantify persisters of *S. aureus* to ciprofloxacin, we measured survival of cultures treated with different concentrations as a function of time (Figure 1). Interestingly, after the initial 2 h of treatment, survival was no longer a monotonic function with respect to ciprofloxacin concentration. Notably, 1 µg/mL ciprofloxacin yielded the lowest survival of any treatment. Concentrations of 5, 10, and 50 µg/mL, which were well above the MIC measured here (0.25–0.5 µg/mL), resulted in higher survival compared to 1 µg/mL that reached up to ~40-fold (10 µg/mL compared to 1 µg/mL at 10 h of treatment, *p*-value < 0.05). Inspection of the survival curves for 5, 10, and 50 µg/mL depicted classic biphasic killing kinetics (Figure 1a), which suggested that treatment times of ≥ 8 h were sufficient for persister measurements. Given such kinetics and the fact that 10 h of treatment yielded the largest difference in survival between the higher concentrations and 1 µg/mL, we chose to focus on the 10 h treatment period for the remainder of this study. Interestingly, survival data showed that the majority of cells that would give rise to persisters at ≥ 5 µg/mL of ciprofloxacin were killed by 1 µg/mL. We note that, at lower ciprofloxacin concentrations, survival from the antibiotic treatment either failed to differentiate from the untreated control (0.1 µg/mL) or at concentrations at the MIC (0.25 and 0.5 µg/mL) cultures rebounded at later times, which could reflect the outgrowth of spontaneously-generated mutants with higher MICs.

### 2.2. Ciprofloxacin Induction of Cell Lysis and Bacteriophage Release is Quenched at High Concentrations

Non-monotonic survival of *S. aureus* when treated with different concentrations of quinolones has been noted previously [30,31]. The most effective concentration was termed the optimum bactericidal concentration (OBC), and reductions in lethality at concentrations greater than the OBC were attributed to quinolone-dependent inhibition of RNA synthesis, because transcription and translation are required for quinolones to exhibit their full bactericidal capacity [24,26,30,31]. More recently, it was found that quinolones can stimulate prophage induction in *S. aureus* at concentrations around their MIC [25,27,32,33]. Given that *S. aureus* NCTC 8325 has three prophages [34] and the OBC that we observed was close to the MIC, we hypothesized that prophage induction might underlie the non-monotonic survival and persister killing we observed.

**Figure 2:**
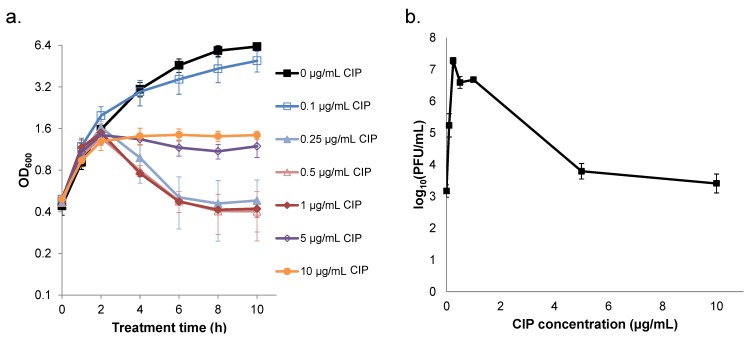
Measurement of cell lysis and bacteriophage release in cultures treated with ciprofloxacin (CIP). *S. aureus* NCTC 8325 was grown in LB for 4 h prior to treatment with 0–10 µg/mL CIP. (**a**) At designated time points, samples were collected for OD_600_ measurements. Data points represent the mean and error bars the SEM of three replicates, except for the untreated control that was replicated twice. (**b**) After 10 h of CIP treatment, samples were filter sterilized and the effluent was assayed for plaque forming units (PFUs). Data points are the mean of the log_10_(PFU/mL) across three replicates, and error bars represent SEM. We note that, for 50 µg/mL CIP, antibiotic present in the effluent interfered with PFU measurements, and this was not observed for treatments of ≤ 10 µg/mL (Figure S1a). Therefore, for consistency between the panels presented here, OD_600_ measurements of 50 µg/mL can be found in Figure S1b.

To test our hypothesis, we monitored cell lysis and bacteriophage release as a function of ciprofloxacin concentration. Using optical density at 600 nm (OD_600_) to measure cell lysis, we observed that lysis occurred with ciprofloxacin concentrations of 0.25, 0.5, and 1 µg/mL as indicated by significant reductions in culture density after 2 h of treatment (all *p*-values < 0.05 for 6, 8, and 10 h samples compared to the 2 h sample for 0.25, 0.5, and 1 µg/mL treatments) (Figure 2a). Alternatively, at concentrations of ciprofloxacin ≥ 5 µg/mL, the OD_600_ of cultures became relatively stable after 2 h of treatment. These results provided evidence that prophage induction occurred at 0.25, 0.5, and 1 µg/mL ciprofloxacin but was suppressed at higher concentrations. To complement the OD_600_ data, we measured bacteriophage release in cultures treated with different concentrations of ciprofloxacin for 10 h. As expected, the highest concentrations of bacteriophage, which were all significantly different from those of the untreated control (*p*-value < 0.05), corresponded to the treatments that exhibited lysis, whereas bacteriophage release from the 5 and 10 µg/mL treatments were quantitatively similar and not significantly different from the untreated control (Figure 2b).

Collectively, these data suggested that prophage induction and cell lysis were responsible for the reduction in survival at 1 µg/mL ciprofloxacin compared to the higher concentrations tested. Further, these data provide support for a mechanism where the non-monotonic survival curve is the result of persister killing that depends on prophage induction.

### 2.3. Prevention of Prophage Induction Protects Persisters and Leads to Monotonic Survival

**Figure 3:**
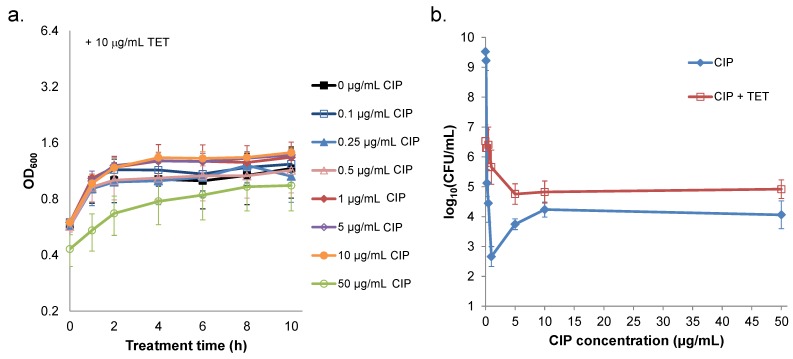
Ciprofloxacin (CIP) treatment of *S. aureus* in the presence of tetracycline. *S. aureus* NCTC 8325 was grown in LB for 4 h and then treated with 10 μg/mL tetracycline (TET), which immediately preceded the addition of 0–50 µg/mL CIP. (**a**) At designated time points, samples were collected for OD_600_ measurements. Data points represent the mean and error bars the SEM of three replicates, except for 50 µg/mL, which was replicated twice. (**b**) After 10 h of treatment with CIP and TET, samples were washed to remove the antibiotics, serially diluted, plated on LB agar, and incubated overnight to enumerate CFUs. For reference, experiments performed without the addition of TET (blue) are depicted in panel (**b**). Data points are the mean of the log_10_(CFU/mL) across three replicates, and error bars indicate SEM. Results from experiments with TET added after 1 h of CIP treatment are provided in Figure S2.

An essential element of prophage induction and lysis is transcription and translation of lytic genes [35,36]. This prompted us to hypothesize that high concentrations of quinolones prevented prophage induction through inhibition of transcription and translation [24,26,31]. If our hypothesis was correct, we reasoned that co-treatment of ciprofloxacin with a translational inhibitor would eliminate cell lysis, produce a monotonic survival curve (remove the trough at 1 µg/mL), and lead to survivals at higher concentrations that were equivalent to those of ciprofloxacin-only treatments. To block translation, we used 10 µg/mL tetracycline, which was sufficient to produce bacteriostasis (Figure 3a and Figure S2a) and impair protein synthesis (Figure S2b). When tetracycline was added immediately preceding ciprofloxacin, cell lysis was eliminated (Figure 3a), and survival became monotonic with significant increases at 1 and 5 µg/mL ciprofloxacin (*p*-values < 0.05) (Figure 3b). Further, co-treatment with tetracycline did not significantly alter survival when 10 and 50 µg/mL ciprofloxacin were used.

In addition, we performed experiments in which tetracycline was added 1 h after treatment with ciprofloxacin (Figure S2), which allowed S. aureus to initiate its normal response to ciprofloxacin prior to translational inhibition. Similar to results obtained with concurrent treatment (Figure 3), addition of tetracycline 1 h after ciprofloxacin treatment prevented cell lysis, eliminated the survival trough at 1 µg/mL ciprofloxacin, and produced comparable killing to ciprofloxacin alone at 50 μg/mL ciprofloxacin (Figure S2). These data demonstrate that translational inhibition can eliminate the prophage-dependent killing of persisters and provide support for a mechanism where increased survival at high quinolone concentrations results from inhibition of protein synthesis.

### 2.4. Quenched Induction of DNA Damage-inducible GFP Reporter at High Ciprofloxacin Concentrations 

**Figure 4:**
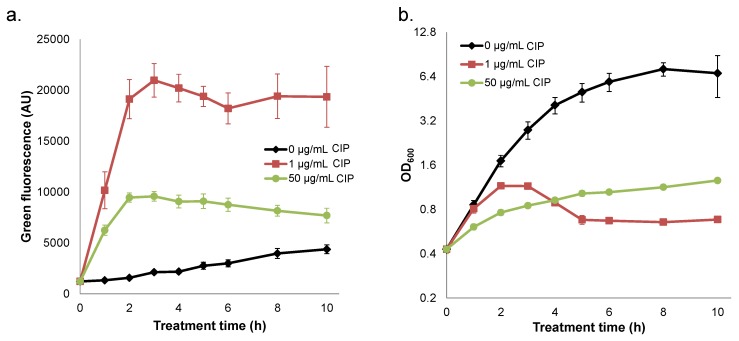
Ciprofloxacin (CIP) treatment of *S. aureus* NCTC 8325 containing a GFP-based SOS reporter. (**a**) Fluorescence from strains carrying pES10 (the P_*recA*_-TIR-*gfpmut2* reporter plasmid) treated with 0, 1, or 50 µg/mL CIP. Prior to fluorescence measurements, the OD_600_ of each sample was adjusted to 0.2. (**b**) OD_600_ measurements corresponding to fluorescence measurements in (**a**). Data are the mean of three or more replicates, and error bars indicate SEM.

Ciprofloxacin has been shown to stimulate prophage induction and the SOS response in *S. aureus* [25,27,32,33]. To provide additional support for a mechanism where ciprofloxacin concentrations greater than the OBC result in enhanced survival due to repression of transcription and translation, we monitored expression from a DNA damage-inducible GFP transcriptional reporter (P_*recA*_-TIR-*gfpmut2*) at both 1 and 50 µg/mL ciprofloxacin; P_*recA*_ is part of the *S. aureus* SOS response that is induced by ciprofloxacin [27]. Expression from the P_*recA*_ reporter was highest under treatment with 1 µg/mL ciprofloxacin and much reduced in cultures treated with 50 µg/mL (Figure 4). We note that fluorescence values from both treatment conditions were considerably higher than those of the untreated control. The reduction in reporter fluorescence at 50 µg/mL compared to 1 µg/mL combined with an insignificant impact of tetracycline on survival at 10 and 50 µg/mL ciprofloxacin (Figure 3) provide support for a mechanism where ciprofloxacin concentrations above the OBC reduce killing by impairing transcription and translation.

### 2.5. Interplay between Prophage Induction and Translational Inhibition by Ciprofloxacin Yields Non-Monotonic Survival

Non-monotonic survival of bacteria treated with antibiotics is not routinely observed [6,9,12,37,38]. However, such behavior had been noted for quinolones previously, and the phenomenon had been attributed to quinolone-dependent inhibition of RNA synthesis at high concentrations, because transcription and translation are needed for quinolones to attain their maximal bactericidal activity [24,26,30,31]. Here, we found that *S. aureus* NCTC 8325 exhibited non-monotonic survival when treated with increasing concentrations of ciprofloxacin, and that this represented an approximate 40-fold loss of persisters at 1 µg/mL ciprofloxacin (Figure 1). Inspired by studies that demonstrated prophage-dependent killing in response to quinolone treatment [23,25], we hypothesized that prophage induction at low concentrations and the deleterious impact of quinolones on transcription and translation at high concentrations [24,26] had combined to produce the non-monotonic survival we observed. In support of this hypothesis, we found that cell lysis and bacteriophage release were maximal at ciprofloxacin concentrations of ~1 µg/mL, whereas cell lysis was not observed for concentrations ≥ 5 µg/mL and bacteriophage release was not significantly different from the untreated control (Figure 2). Further, we observed that blocking prophage induction with tetracycline, either at the same time as ciprofloxacin treatment or 1 h afterward, removed the trough in survival at 1 μg/mL, yet failed to impact survival at higher concentrations (Figure 3 and Figure S2). In addition, we observed that a high concentration of ciprofloxacin exhibited attenuated expression of GFP from a DNA damage-inducible transcriptional reporter when compared to 1 µg/mL (Figure 4). Collectively, these data provide evidence for a mechanism where the killing of *S. aureus* persisters at 1 µg/mL ciprofloxacin is dependent on prophage induction.

Whether prophage that were endogenous to persisters were responsible for the killing or whether it was associated with super-infection of persisters with bacteriophage released from dead cells was not determined here. In a previous study, using a high persistent mutant of *Escherichia coli* (*hipA7*), Pearl and colleagues found that the physiology of persisters to ampicillin protected them from endogenous prophage induction but did not render them immune to cell death caused by super-infection [39]. The extent to which such phenomena apply to different bacteria and persisters to different antibiotics remains to be determined; however, we note that delineating the source of bacteriophage responsible for the persister killing observed here is an interesting topic for a future study. In addition, delineating the roles of mediators of prophage induction (e.g., RecA [40,41]) and persistence (e.g., TA modules [42,43,44]) could provide further insight into the phenomenon described here. 

## 3. Experimental Section

### 3.1. Bacterial Strains, Chemicals, and Media

Strains used in this study are listed in Table 1. All chemicals were purchased from Thermo Fisher Scientific (Waltham, MA, USA) or Sigma-Aldrich (St. Louis, MO, USA). LB medium (10 g/L tryptone, 5 g/L yeast extract, and 10 g/L NaCl) was used for planktonic growth, and LB agar (LB + 15 g/L agar) was used to measure CFUs. Ciprofloxacin was dissolved in 0.2 N HCl, stock solutions were adjusted to 0.04 N HCl and sterilized with a 0.22 µm syringe filter the same day as experiments. Stock solutions of tetracycline were dissolved in deionized MilliQ water and sterile filtered; erythromycin (ERM) was dissolved in ethanol. Phage buffer, top agar, bottom agar, and CYGP used in phage transduction and for phage assays were described previously [45].

**Table 1:** Strains and plasmids used in this study.

Strains/Plasmids	Description	Source
Bacterial Strains:		
*S. aureus* 8325	Wild-type strain	[46]
*S. aureus* 8325 pES10	Wild-type strain harboring pES10	This study
*S. aureus* RN4220	Cloning strain derivative of *S. aureus* NCTC 8325	Lab of Thomas Muir
*S. aureus* RN10395	80α lysogen used for phage transduction to facilitate plasmid transformation	Lab of Richard Novick
*E. coli* SA08B	Cloning strain for plasmid construction	Lucigen
Plasmids:		
pCN51	pCN shuttle vector (P_*cad*_-*cadC*), *ermC* (*S. aureus*), *amp* ColE1 ori (*E. coli*)	Lab of Thomas Muir
pUA66	Plasmid source of *gfpmut2*	[47]
pES1	pCN51 with *gfpmut2* (*gene 10* RBS)	This study
pES4	pES1 with MCS replacing P_*cad*_-*cadC*	This study
pES5	pES4 with *gfpmut2* (TIR RBS)	This study
pES10	P_*recA*_-TIR-*gfpmut2*	This study

### 3.2. Fluorescent Reporter Construction

Generalized cloning procedures are as follows: DNA inserts were PCR amplified from plasmid or chromosomal DNA with Phusion DNA Polymerase (New England Biolabs, Ipswich, MA, USA). Inserts and vectors were digested with restriction enzymes (New England Biolabs) according to the manufacturer’s protocols, and the vectors were treated with Antarctic Phosphatase (New England Biolabs) to circumvent self-ligation before the inserts and vectors were ligated together using Quick Ligase (New England Biolabs). Ligation products were transformed into chemically competent *E. coli* SA08B prepared using the Mix and Go *E. coli* Transformation Kit (Zymo Research, Irvine, CA, USA) and transformants were plated immediately on LB agar supplemented with 100 µg/mL ampicillin and incubated overnight at 37 °C. Successful ligation was confirmed by colony PCR and DNA sequencing (Genewiz, South Plainfield, NJ, USA). *S. aureus* genomic DNA used as the PCR template was extracted using a DNeasy Blood and Tissue Kit (Qiagen, Valencia, CA, USA) following the manufacturer’s instructions. As *S. aureus* is a Gram-positive organism, the lysis step was modified such that the cells were incubated in 500 µg/mL lysostaphin, 20 mM Tris-HCl, 2 mM EDTA (pH 8.0), and 1.2% Triton X-100 at 37 °C for 1 h; Proteinase K was added and the sample was incubated at 56 °C for an additional 2 h. Plasmids were extracted using a QIAprep Spin MiniPrep Kit (Qiagen).

pCN51 is a high copy *E. coli*-staphylococcal shuttle vector with a cadmium-inducible cassette P_*cad*_-*cadC* [48], and it was used as the backbone of the P_*recA*_ reporter plasmid, pES10. The primers used for constructing pES10 are listed in Table 2. To construct this reporter plasmid, we first cloned *gfpmut2,* derived from pUA66 [47], into pCN51 at KpnI and EcoRI restriction sites so that it was positioned after P_*cad*_-*cadC* (generating pES1); primers used to amplify *gfpmut2* were designed so that a *gene10* ribosomal binding site (RBS) was inserted upstream of *gfpmut2*. In order to generate a versatile backbone, we then synthesized a multiple cloning site by the extension of two overlapping oligonucleotides using the Klenow fragment (Thermo Fisher Scientific, Waltham, MA, USA), and we replaced P_*cad*_-*cadC* of pES1 with this MCS (using the SphI and KpnI restriction sites) (generating pES4). In work unrelated to the data presented above, replacement of the cadmium cassette of pES1 with different promoters failed to generate a high fluorescence signal (data not shown). Low fluorescence of staphylococcal reporters has been reported to be highly sensitive to the RBS region when compared within the same backbone, with the RBS regions ranked as *gene10 < sarA < sod < hld* with respect to fluorescence output [49]. In a later report, the translation initiation region (TIR) reported to enhance fluorescence in *E. coli* [50] was found to improve fluorescence output compared to the *sod* RBS region in the staphylococcal pCN vectors [51]. Thus, the *gene10* RBS region was replaced with the TIR sequence (TIR: 5′-TGATTAACTTTATAAGGAGGAAAAACATATG-3′), generating pES5. Finally, P_*recA*_, consisting of 505 bp upstream of the start codon of *recA*, was amplified from *S. aureus* genomic DNA and ligated at SbfI and KpnI sites of pES5 to generate pES10.

**Table 2:** Primers used in plasmid construction.

Primer	Sequence
gfpmut2_FW_KpnI + 3stop_2	5′-CTGACAGGTACCGTTAACTAATTAATTTAAGAAGGAGATATACATATGAG-3′
gfpmut2 + EcoRI_REV	5′-AGCATAGAATTCTTATTTGTATAGTTCATCCATGCCA-3′
MCS_FW	5′-TCATCATGGTACCCGGGGATCCTCTAGAGTCGACCTGCAGGGCATGC-3′
MCS_REV	5′-CTAGTAGGCATGCCCTGCAGGTCGACTCTAGAGGATCCCCGGGTACC-3′
gfpmut2_KpnI_TIR_FW	5′-CATCATGGTACCTGATTAACTTTATAAGGAGGAAAAACATATGAGTAAAGGAGAAGA-3′
SbfI_PrecA_FW	5′-TAGTAGCCTGCAGGATGATGGTATTACTAATGGTGC-3′
KpnI_PrecA_REV	5′-TAGTAGGGTACCAGCGAGACCTCCTAATTGAAATTGCTA-3′

Vectors were maintained in the cloning strain *E. coli* SA08B (Lucigen, Middleton, WI, USA), which was designed with a methylation pattern to allow direct transfer of DNA from *E. coli* to wild-type *S. aureus* strains. However, we were unsuccessful in directly transforming intact plasmids from *E. coli* SA08B to *S. aureus* NCTC 8325, and therefore, we used *S. aureus* RN4220 as a cloning intermediate. Sequence-verified plasmids from *E. coli* SA08B were transformed into electrocompetent *S. aureus* RN4220 cells following electroporation protocols described previously [52], and transformants were selected on LB agar containing 10 µg/mL ERM. The *S. aureus* 80α lysogen RN10395 was used to facilitate plasmid transfer from RN4220 to NCTC 8325, as phage 80α is a helper phage that enhances competence for transformation of the strain bearing it [53]. Transduction and transformation was performed using media and methods described previously, with slight modifications [34,45]. In order to induce 80α phage from the transducing strain, RN10395 was grown to mid-exponential phase in 10 mL of CYGP broth at 37 °C with shaking, centrifuged, and resuspended in the same volume of phage buffer [45]. The culture was transferred to a Petri dish, which was placed on a rocking table, and the culture was irradiated with an overhead UV lamp for 20 s. An equal volume (10 mL) of CYGP was added to the culture, and the culture was incubated with slow shaking (75 rpm) at 30 °C for ~2 h until lysis occurred. The sample was filter sterilized, and the lysate was stored at 4 °C. Next, the RN4220 donor strain containing the plasmid of interest was grown to mid-exponential phase in 10 mL of CYGP broth. Aliquots (1 mL) were transferred to a 6-well plate and diluted 1:1 with phage buffer. Wells were treated with various volumes of phage lysate (5–100 µL) and incubated with slow shaking for ~2 h at 30 °C as was performed for the 80α lysogen. The well with the lowest volume of added phage lysate that achieved lysis of the culture was filter sterilized with a 0.22 µm syringe filter, and this infective lysate was stored at 4 °C. Finally, 500 µL of an exponential culture of the recipient strain, NCTC 8325, grown in CYGP was combined with 5 µL of 1 M CaCl_2_ and 25 µL of the infective lysate and incubated for 15–20 min at room temperature. Then, 100 µL of 1 M sodium citrate was added, and the culture was incubated for 0.5–1 h at 37 °C. The culture was added to 3 mL of melted top agar, mixed gently, and poured immediately on a phage agar plate containing 10 µg/mL ERM. When solidified, plates were incubated at 37 °C overnight. Single colonies were inoculated into 3 mL LB containing 10 µg/mL ERM, grown overnight, mixed with equal volumes of LB-glycerol and stored as a −80 °C stock. Function of the plasmids in each clone was confirmed by fluorescence enhancement following CIP treatment compared with strains harboring the promoterless control vector, pES5.

### 3.3. Measurement of MIC

Quantifications of MICs were conducted in LB in 96-well plates using a procedure described previously [54]. Briefly, concentrated antibiotic stocks were added to LB and a 2-fold dilution was performed in LB across the plate. A *S. aureus* overnight culture grown in LB at 37 °C was diluted to ~10^5^ CFU/mL and added to the antibiotic dilution series at a 1:1 ratio. The 96-well plate was then incubated statically at 37 °C overnight, and the MIC was defined as the lowest antibiotic concentration inhibiting visible growth. Interestingly, in a number of these plates, biofilm formation was observed in the well just below the MIC. All lower concentrations had turbid wells with no biofilm colonies when the liquid was removed; however, just below the MIC, the bulk fluid lacked turbidity, but defined biofilm colonies were visible on the bottom of the well. When observed, this was still considered growth, and the MIC was classified as one concentration above that well.

### 3.4. Antibiotic Treatment Assays

*S. aureus* NCTC 8325 from a −80 °C stock were used to inoculate 3 mL of LB, and the culture was grown for 16 h at 37 °C and 250 rpm. Overnight cultures were diluted to an OD_600_ = 0.01 in 50 mL of LB in 500 mL baffled flasks and grown at 37 °C and 250 rpm. At OD_600_ ~0.4–0.6, 10 mL aliquots were transferred to 250 mL baffled flasks. When more than 50 mL of culture was required, identical 50 mL cultures were pooled prior to distribution of 10 mL aliquots. Where applicable, tetracycline was added to cultures at a final concentration of 10 µg/mL immediately prior to or 1 h following treatment with ciprofloxacin. Ciprofloxacin was added to cultures at final concentrations ranging from 0–50 µg/mL. At designated time points, samples were removed for persister enumeration and OD_600_ measurements. For cell counts, samples were removed (200 µL to 1 mL depending on the time point), and the volume was brought up to 1 mL with phosphate-buffered saline (PBS) as needed. To remove the antibiotic, cells were collected by centrifugation (3 min, 21,000 × *g*), 900 µL of the supernatant was removed, 900 µL of PBS was added, the cell pellet was resuspended, and these steps were repeated a second time. Samples were then serially diluted in PBS, and 10 µL spots were plated on LB agar. To improve the limit of detection, 100 µL spots were plated in some cases. Plates were incubated at 37 °C for ~20 h, and persisters were enumerated by counting CFUs. The limit of detection (1 colony in the zero dilution of a 100 µL spot) was 1.7 log_10_(CFU/mL). To measure cell density, samples were removed from flasks, and OD_600_ was measured with a Synergy H1 Hybrid Multi-Mode microplate reader (BioTek, Winooski, VT, USA).

### 3.5. Plaque Assays

Phage buffer, top agar, and bottom agar were prepared as previously described [45]. To obtain sample lysates to enumerate plaque forming units (PFUs), *S. aureus* NCTC 8325 cultures were filtered with 0.22 µm syringe filters, and the effluent were stored at −20 °C until subsequent analysis. Since different concentrations of ciprofloxacin in the different sample lysates could have impacted PFU measurements, all undiluted lysates were adjusted to contain the same ciprofloxacin concentration, which was 10 µg/mL. Control experiments with samples whose ciprofloxacin concentrations were not adjusted in this manner demonstrated that this step did not alter PFU measurements (Figure S1a). 10-fold dilutions of lysates were prepared using phage buffer. A 50 µL aliquot of each diluted lysate was added to a 0.5 mL aliquot of RN4220 at an OD_600_ ~0.6, which had been inoculated from a −80 °C stock into 25 mL of CYGP and grown for 6–8 h. The lysate/RN4220 mixture was incubated at 37 °C for 20 min. Then, the lysate/RN4220 mixture was added to 5 mL of melted phage top agar and mixed well before transfer to the presolidified phage bottom agar plate. The plate was tilted to evenly spread the lysate/RN4220/top agar. After the top agar had solidified, the plate was grown at 37 °C overnight (~16 h), after which phage plaques were enumerated.

### 3.6. Fluorescence Assays

The same protocol used for the antibiotic treatment assays described above was used for fluorescence assays of *S. aureus* NCTC 8325 containing pES10 with the addition of 10 µg/mL ERM for plasmid retention. At designated time points, samples were removed, the cells were collected by centrifugation (3 min, 21,000 × *g*), the media was removed, and the remaining cell pellet was resuspended in 320 µL of PBS. The OD_600_ of each sample was adjusted to 0.2 in PBS in a total volume of 300μL and GFP fluorescence was measured in black, clear bottom 96-well plates using 485/515 nm excitation/emission on a Synergy H1 Hybrid Multi-Mode microplate reader (BioTek). Fluorescence values of equivalent volumes of PBS were used as blanks.

### 3.7. Statistical Analyses

The statistical significances reported were determined using two-tailed Student’s *t*-tests with unequal variances and the null hypothesis that the means of the two samples were equivalent. *p*-Values less than 0.05 were considered significant.

## 4. Conclusions

Since the majority of *S. aureus* strains harbor prophage [32] and non-monotonic survival with quinolone treatment had been previously observed for different staphylococci [30,31], we postulate that the phenomenon of decreased persister survival due to prophage induction at low ciprofloxacin concentrations investigated here may be broadly relevant. However, this knowledge does not necessarily provide clear indications of what quinolone concentrations should be used to fight *S. aureus* infections. At ciprofloxacin concentrations around the OBC, survival of *S. aureus* is severely compromised (Figure 1), but bacteriophage concentrations increase dramatically (Figure 2). Given that bacteriophage are known to transfer pathogenicity islands and other virulence determinants [32,33], it may be more desirable to use higher ciprofloxacin concentrations, which result in higher survival of *S. aureus* but significantly limit induction of prophage.

## Supplementary Materials

Supplementary materials can be accessed at: http://www.mdpi.com/1424-8247/8/4/778/s1.
