# Supplementary material for: Non-Monotonic Survival of Staphylococcus aureus with Respect to Ciprofloxacin Concentration Arises from Prophage-Dependent Killing of Persisters
Source: Pharmaceuticals (Basel). 2015 Nov 17;8(4):778–92. doi: 10.3390/ph8040778 (PMC4695809; doi:10.3390/ph8040778)
Supplement: Supplementary file 1 [file pharmaceuticals-08-00778-s001.pdf]

Supplementary Materials

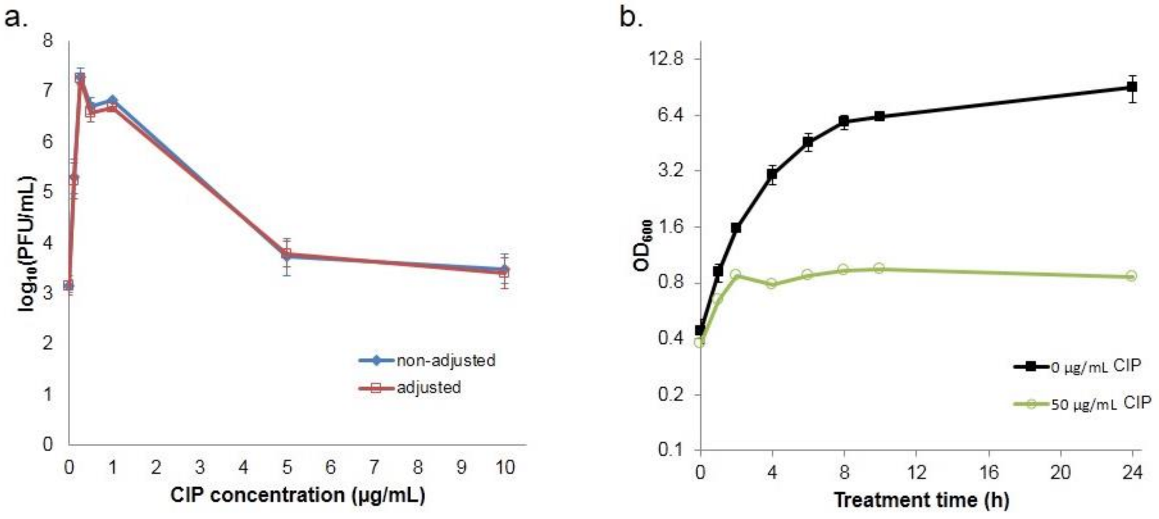

Figure S1. Supplemental measurements of bacteriophage release and lysis in cultures treated with ciprofloxacin (CIP). *S. aureus* NCTC 8325 was grown in LB for 4 hours prior to treatment with 0–10 µg/mL CIP. (**a**) After 10 h of treatment, samples were filter sterilized, and the effluents were assayed for plaque forming units (PFUs). To assess whether the different concentrations of CIP in the effluents impacted the PFU results, effluents whose concentrations were adjusted to 10 µg/mL were compared to those of non-adjusted controls. As indicated, PFU measurements were the same. Data points are the mean of the $\log_{10}$(CFU/mL) across three replicates, and error bars represent SEM. (**b**) At designated time points, samples were collected for OD_{600} measurements. We note that the 50 µg/mL data represents one replicate.

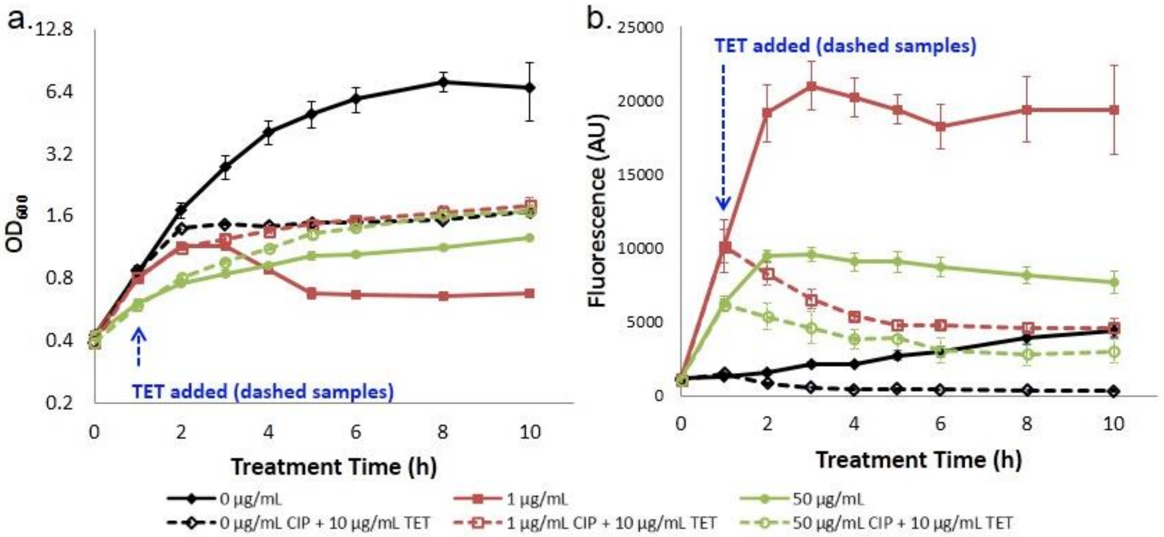

Figure S2. *Cont.*

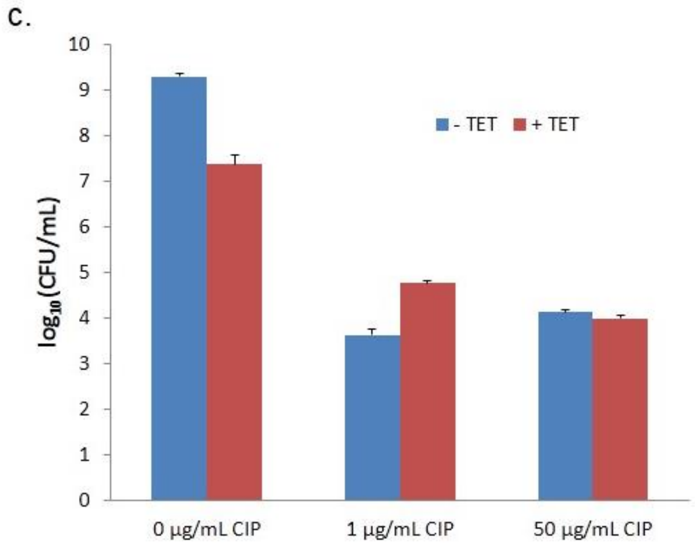

Figure S2. Impact of tetracycline on growth, prophage induction, protein synthesis, and survival after ciprofloxacin (CIP) treatment of *S. aureus* NCTC 8325 harboring pES10 (P_{recA}-TIR-*gfpmut2* reporter plasmid). (**a**) Optical density and (**b**) fluorescence measurements after ciprofloxacin treatment of exponential phase cultures. For indicated samples (dashed), tetracycline (TET) was added 1 h after ciprofloxacin. (**c**) log_{10}(CFU/mL) after 10 h of treatment with CIP at indicated concentrations. We note that at 0 μg/mL CIP the sample without TET continued to grow far beyond the density of the sample with TET. Fluorescence, which is reported in arbitrary units (AU), was measured from samples after adjustment to an OD_{600} of 0.2. Data are the mean of three experiments, and error bars indicate SEM.
